# Ecosystem services in coupled social–ecological systems: Closing the cycle of service provision and societal feedback

**DOI:** 10.1007/s13280-015-0651-y

**Published:** 2015-05-12

**Authors:** Michael Nassl, Jörg Löffler

**Affiliations:** Department of Geography, University of Bonn, Meckenheimer Allee 166, 53115 Bonn, Germany

**Keywords:** Human–nature interactions, Ecosystem services, Driver-Pressure-State-Impact-Response (DPSIR) model, Mountain Research, Sierra Nevada (Spain)

## Abstract

Both the ‘cascade model’ of ecosystem service provision and the Driver-Pressure-State-Impact-Response framework individually contribute to the understanding of human–nature interactions in social–ecological systems (SES). Yet, as several points of criticism show, they are limited analytical tools when it comes to reproducing complex cause–effect relationships in such systems. However, in this paper, we point out that by merging the two models, they can mutually enhance their comprehensiveness and overcome their individual conceptual deficits. Therefore we closed a cycle of ecosystem service provision and societal feedback by rethinking and reassembling the core elements of both models. That way, we established a causal sequence apt to describe the causes of change to SES, their effects and their consequences. Finally, to illustrate its functioning we exemplified and discussed our approach based on a case study conducted in the Alpujarra de la Sierra in southern Spain.

**Electronic supplementary material:**

The online version of this article (doi:10.1007/s13280-015-0651-y) contains supplementary material, which is available to authorized users.

## Introduction

Human well-being is considerably threatened by increasing damage to or losses of natural resources. These resources and their users are embedded in complex social–ecological systems (SES) (Ostrom 2009). In order to develop policies enhancing the sustainability of SES and thus safeguarding the livelihoods of their affiliated users, frameworks providing an adequate overview of the problems, associated causes, and resulting effects are needed. Such frameworks help to “organize diagnostic, descriptive, and prescriptive inquiry” (McGinnis and Ostrom 2014, p. 30). Thus, they are useful tools to create a common vocabulary of concepts and terms, integrate causal explanations provided by different theories, and facilitate the development of models to explain processes and predict outcomes (McGinnis and Ostrom 2014).

In a SES, ecological subsystems such as a resource system interact with resource users and their governance systems to generate outcomes at the SES level (Berkes and Folke 1998; Ostrom 2009). To capture the outputs of the ecological subsystem (and their values) more comprehensively than the notion of a ‘resource’ does, the concept of ‘ecosystem services’ (ES) was developed in the late 1990s (Costanza et al. 1997; Daily 1997). The idea behind ES describes how nature supports human well-being by generating multiple benefits (Costanza et al. 1997; Daily 1997; MA 2005). ES quickly gained considerable attention among scientists and practitioners, but they also encountered multifaceted dissent. The most recurring objections to the ES idea include its ‘anthropocentric focus’ (Sagoff 2008) and its ‘focus on monetary valuation’ (Gómez-Baggethun and Ruiz-Pérez 2011), the fear that it promotes an ‘exploitative human–nature relationship,’ and the ‘commodification of nature’ (Fairhead et al. 2012). Furthermore, some critics hold the concepts ‘normative nature’ against it, which, according to them, implies that all outcomes of ecosystem processes are positive, while ignoring ‘ecosystem restraints’ (Sagoff 2002; McCauley 2006). Finally, prevailing definitions, typologies, and terminologies lack consensus (see Boyd and Banzhaf 2007; Wallace 2007; Costanza 2008; Fisher et al. 2009; Potschin and Haines-Young 2011).

To depict the causal chain along which ES evolve from natural structures and processes within the resource system until they generate gains in human well-being, Haines-Young and Potschin (2010) presented the ‘ecosystem service cascade.’ The ES cascade, in its conciseness, has proven as a very useful scheme to allocate and define the basic elements of ES generation and delivery. However, the framework has been criticized for neglecting societal ‘input’: be it the underrepresentation of societal feedback mechanisms, the influence of land-use on ES provision (e.g., van Oudenhoven et al. 2012), or the disregard of human involvement as an essential part of the ‘cascade process’ itself (Spangenberg et al. 2014).

To understand the causes and effects of human-induced damages and modifications to the resource system that provides ES, it is necessary to position the ‘cascade’ in a broader cause–effect scheme within a SES. Therefore, several authors have presented frameworks that integrate ES into the Driver-Pressure-State-Impact-Response (DPSIR) scheme (e.g., Vandewalle et al. 2009; Rounsevell et al. 2010; Müller and Burkhard 2012; van Oudenhoven et al. 2012; Helming et al. 2013; Kandziora et al. 2013; Kelble et al. 2013). The EEA formulated the DPSIR framework to identify and structure indicators for the causes and effects of human-induced changes to the environment (EEA 1995; Burkhard and Müller 2008). The DPSIR facilitates the analysis of complex SES by simplifying and qualitatively describing their inherent cause–effect relationships and thereby supports environmental decision-making (Turner et al. 1998; Burkhard and Müller 2008; Potschin 2009). While its didactic clarity underpins its applicability and partly explains its significant resonance in scientific literature as well as its appeal to environmental policy and practice, it has also drawn increasing skepticism (see e.g., Svarstad et al. 2008; Maxim et al. 2009; Potschin 2009; Spangenberg et al. 2009; Kelble et al. 2013). Its simplicity can hinder the framework from reproducing the complexity of the real world and therefore challenges its value as an analytical tool (Maxim et al. 2009). Furthermore, Svarstad et al. (2008) argue that it fails to generate neutral knowledge, as it favors conservationist positions while neglecting other stakeholders’ perspectives. As the DPSIR focuses on human-induced changes to the environment, it disregards important non-human drivers of change (Svarstad et al. 2008; Potschin 2009). Moreover, its impact component predominantly accounts for negative consequences of human activities and therefore does not facilitate proactive management practices (Kelble et al. 2013). In response to these shortcomings, some scientists came up with conceptual improvements: Maxim et al. (2009) advocated the coupling of the framework with the ‘four spheres of sustainability,’ whereas Niemeijer and de Groot (2008) proposed to expand DPSIR’s causal chain to a ‘causal network.’

The DPSIRs strength lies in identifying and describing the causes and effects of human-induced changes to the environment, whereas the ES cascades’ asset consists in causally linking the environment and its structures to the fundaments of society (i.e., human well-being). Thus, we argue, by combining the two frameworks, they can mutually enhance their comprehensiveness and overcome their individual ‘flaws.’ Based on these considerations, we present an integrated framework, which—with some essential modifications—combines the core components of the DPSIR and the cascade with the objective to embed ES in a broader SES context. It is geared towards the aim, to analytically describe the interactions between the ecological subsystem and social users and their governance systems between nature and society and how these ‘shape’ each of those subsystems of a SES. This entails a range of more detailed focal questions: What are the natural or human-induced causes of undesired or desired outcomes of social–ecological interactions? In what ways do these underlying causes affect the societal and natural subsystem? What are the systems’ responses to alterations? How is the interaction of ecological *and* social factors expressed in the provision of ES? What influence has human involvement in the generation of ES?

In the remainder of this paper, we identify and discuss the components of the ES cascade and the DPSIR framework and lay out the underlying assumptions and findings regarding the merging of the two frameworks. After presenting and outlining the new framework itself, we continue with an exemplary exercise—carried out within the SES of the *Río de Mecina* valley (Spain)—to demonstrate the functioning of our framework. Finally, we conclude with a discussion of the added value of our framework underpinned by some aspects of our case study.

## Merging the cascade and the DPSIR

### The ‘cascade’ and its components

**Fig. 1:**
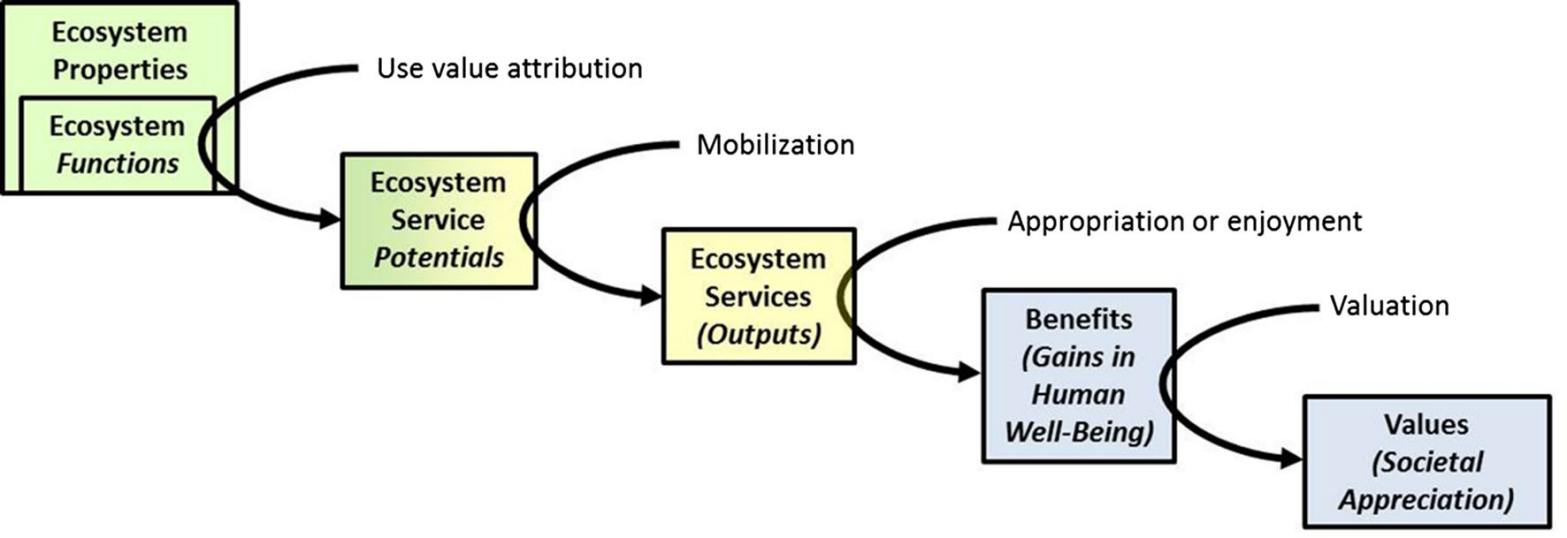
The ecosystem service cascade (modified after Spangenberg et al. 2014, based on Haines-Young and Potschin 2010). *Arrows* describe human involvement in the process of ES generation and delivery: Use value attribution turns biophysical ecosystem functions into ES potentials, which, when mobilized, provide ES. Through appropriation or enjoyment, ES then generate benefits to human well-being. These benefits manifest in societal (or economic) values after economic or non-economic valuation. *Blue colors* indicate the anthroposphere, *green colors* indicate the biosphere, and *yellow colors* indicate the sphere of overlapping (i.e., social–ecological interactions)

The ‘ecosystem service cascade’ by Haines-Young and Potschin (2010, p. 116) connotes that ES are part of a type of “production chain” that links an ecosystem’s biophysical structures and to socio-economic or cultural gains in human well-being (see Fig. 1). One end of the chain represents these structures and processes (termed *ecosystem properties* herein). The other end represents the contribution of the ecosystem to human well-being (*benefits*). Because such *benefits* can be valued differently by different people (or not valued at all), most adaptations of the cascade separate *values* from *benefits* and position them at the end of the chain (TEEB 2010; Potschin and Haines-Young 2011).

The logic of an ES production chain implies that between *ecosystem properties* and *benefits*, there must be at least one intermediate link, namely ES themselves. However, under such rigid categorization, supposed services like ‘primary production’ or ‘nutrient cycling’ do not connect directly to a benefit—they rather contribute indirectly to human well-being via another service (e.g., ‘the provision of crops’). Based on this consideration, the integration of another distinct link between ecosystem properties and ES, called *ecosystem functions*, became prevalent (Daily 1997; de Groot et al. 2002; Boyd and Banzhaf 2007; Fisher et al. 2009). To distinguish them from natural processes in general, they can be defined as the ‘capacity of natural processes and structures to provide services’ (see de Groot et al. 2002; Haines-Young and Potschin 2010) and they are “best conceived as a subset of ecological processes and ecosystem structures” (de Groot et al. 2002, p. 394). In the original depiction of the cascade, ecosystem functions then give rise to ES through human utilization, which in turn then generate a benefit (Haines-Young and Potschin 2010). Because of its importance to monetary valuation of ES, the distinction between ES and benefits is a vexed issue (e.g., Boyd and Banzhaf 2007; Wallace 2007; Costanza 2008). However, if regarded as the end-link of a chain that connects ecosystem structures and processes to human well-being, benefits must provide direct gains for the latter. In other words, while the existence of, for example, timber alone is not a benefit, it becomes beneficial in the form of shelter when used to build a home.

With ecosystem properties, functions, services, benefits, and values, the basic ‘steps’ of ES generation and delivery are set. However, in that original form, the framework does not satisfyingly describe the dynamics and changes *between* those steps—the arrows connecting the cascade elements stay unlabeled. Spangenberg et al. (2014) extensively addressed that weakness of the framework and we will follow their reinterpretation of the cascade (see Fig. 1). Their main argument is to acknowledge the human involvement within each cascade step: The ecological processes and ecosystem structures that form ecosystem functions exist regardless of whether humans consume, appreciate, or value their ‘output.’ To generate an ES, human involvement is necessary. Humans need to realize the potential usefulness of a certain ecosystem function to their well-being. Spangenberg et al. (2014, p. 25) call this step “use value attribution.” Use value attribution transforms ecosystem functions into *ecosystem service potentials*. To avoid confusion, they use the term ecosystem function in a strict bioscience sense (i.e., as natural processes that operate within an ecosystem) and therefore depict it in one ‘box’ with ecosystem properties (see Fig. 1). In order to generate the actually available ES, again human intervention is required: Humans must mobilize ES potentials, in many cases through the investment of labor, resources, knowledge, and time (Spangenberg et al. 2014). Finally, by appropriating (e.g., harvesting, hunting), through enjoyment or other forms of consumption of the ES, it provides the benefit.

Despite its obvious temporal progression, the cascade is a rather static conceptualization. It describes the supply/demand complex only within a certain period (i.e., from provision to consumption) and unidirectional (i.e., from the ecosystem to society). It largely discounts feedbacks from the end of the causal chain to its beginning. It mentions pressures affecting ecosystem structures and the possibility of policy actions to limit them, but does not elaborate the topic. Thus, besides the modifications by Spangenberg et al. (2014), further adjustment is necessary to account for the complexity of real SES. To integrate underlying causes of human involvement in the cascade, as well as consequential adaptation to environmental changes, we propose to merge it with the DPSIR framework.

### The DPSIR framework

Under the classic interpretation of this model, *drivers* exert *pressures* on the environment and thereby change its *state* (Smeets and Weterings 1999). This altered state has an *impact* on “human health, ecosystems and materials” (Smeets and Weterings 1999, p. 6) and leads to a societal *response*. The societal response in turn feeds back on all other components (Smeets and Weterings 1999; Niemeijer and de Groot 2008).

*Drivers* (or driving forces) are “factors that cause changes or lead the behavior of a system” (Burkhard and Müller 2008, p. 968). Maxim et al. (2009) remarked that the majority of publications only considered anthropogenic factors as drivers. As a result, the DPSIR often ignores key non-human drivers of environmental change (Svarstad et al. 2008; Potschin 2009). Some modified applications of the DPSIR (e.g., MA 2005; TEEB 2010) differentiate between direct and indirect drivers: Direct drivers explicitly influence the system, while indirect drivers act by changing the conditions of one or more direct drivers (Burkhard and Müller 2008). Furthermore, drivers can be exogenous or endogenous to a system, depending on the scale of the system under consideration. Svarstad et al. (2008) identify two scales, which the system a DPSIR framework describes is bounded by: the scale, defined by the drivers (i.e., macroscale) and the scale at which impacts occur (i.e., mesoscale). If these boundaries coincide, the driver is endogenous, yet in most cases, drivers or responses that act at one scale will determine impacts on a different scale and thus these drivers are ‘external.’

While drivers are the underlying causes of change, a *pressure* is the actual stimulus that alters the state of the system and hence induces impacts. In the standard interpretation of the model, pressures are mainly the consequence of human-induced actions (Burkhard and Müller 2008). This again poses a problem when including non-human-induced causes of change such as natural climatic variability. Is a drought in consequence of climatic variability to be recognized as a pressure affecting the state of the ecosystem? This question also comprises a general difficulty of how to define pressures. Under which circumstances do general influencing factors become stressors to the environment? Svarstad et al. (2008), for example, have shown that the perception of the presence of a pressure strongly depends on the system of knowledge and belief of the stakeholders involved. Furthermore, definitions differ in respect to the object of change that a pressure initiates: Whereas some authors regard any human influence on the environment as negative and thus as a pressure, some studies draw a line and consider only changes beyond that threshold as ‘negative enough’ to count as pressures (Maxim et al. 2009). A third variant is to define pressures by their impact, i.e., only changes with a negative impact are considered pressures (Maxim et al. 2009).

The *state* component of the DPSIR describes the altered conditions of the environment. Changes in the environmental state are often delayed reactions to pressures that occurred in the past, yet, depending on the pressure exerted, they also can be abrupt. In many cases, altered natural conditions will have an *impact* on society, as most components of human well-being depend largely on an intact environment. Kelble et al. (2013, p. 2) argue that impacts, in the standard DPSIR interpretation, are “unavoidably” considered negative. This, they continue, leads to a focus upon responses to these adverse impacts, rather than “proactive management to sustain and maximize ecosystem services” (Kelble et al. 2013, p. 2).

Impacts on human well-being trigger societal *responses* in the form of human actions taken to intervene in the process. These interventions may address the driver, the pressure, or the impact itself. Typical instruments of responses include laws (e.g., bans or production standards), landscape or construction planning, or economic or market instruments (Burkhard and Müller 2008).

### Closing the cycle of ecosystem service provision and societal feedback

**Fig. 2:**
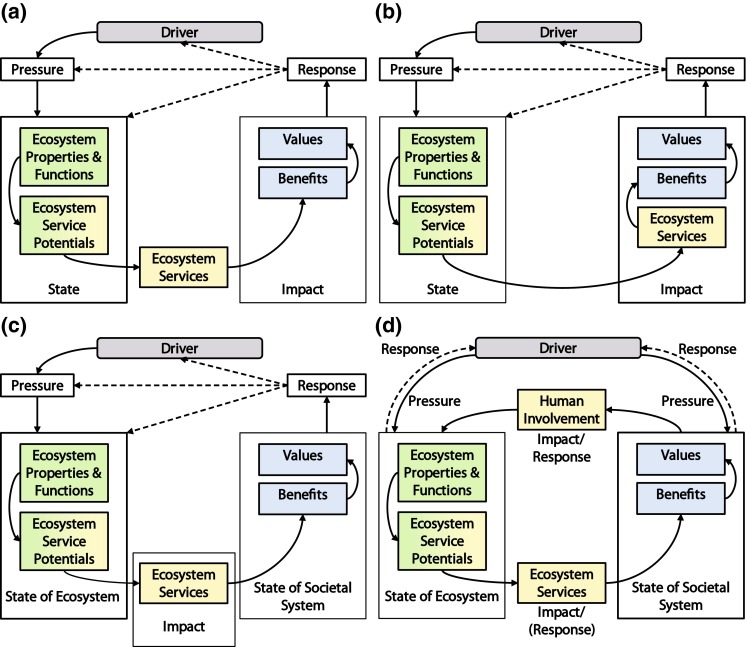
**a**–**d** Merging the cascade and the DPSIR to create the cycle of ecosystem service provision and societal feedback. We assigned ecosystem properties, ecosystem functions, and ES potentials to the DPSIR component *State*, based on the perception, that they are essential variables describing the state of the ecosystem. Kandziora et al. (2013) leave ecosystem services as an intermediate step between the *State* (of the ecosystem) and the ecological *Impact* on society (**a**). However, we argue that ecosystem services are *a means of impact*, rather than its cause and therefore allocate them to the DPSIR component *Impact* (**b**). Benefits and values are considered societal phenomena caused by that impact. They describe the state of the societal system in relation to ecosystem service delivery. Thus, we introduced a second state component (**c**). To acknowledge the human involvement in ecosystem service supply and delivery, we then established a second causal chain linking the two state components, closing the causal circle (**d**)

Although the ES concept and DPSIR framework in some parts coincide, various structural mismatches become apparent when merging them. Some authors equated the DPSIR component state to the whole ES provision–supply complex (e.g., RUBICODE’s ‘coupled DPSIR and SES framework’; see Vandewalle et al. 2009; Rounsevell et al. 2010). From this perspective, state does not represent the state of the “environmental conditions” sensu OECD (1997, p. 12), but rather the state of all “elements relevant to the demand and supply of the ecosystem service” (Vandewalle et al. 2009, p. 41). This would mean allocating the whole service cascade to this DPSIR component. In later publications, only ecosystem properties and ecosystem functions are equated with the component state (e.g., Müller and Burkhard 2012; Kandziora et al. 2013). This approach, however, raises the question of where ‘to fit in’ ES potentials, benefits, and ES. Spangenberg et al. (2014) emphasize that ES potentials are human constructs. As such, it would be logical to assign them somewhere in between the ecosphere and the anthroposphere. However, ES potentials are an essential variable in analyzing the conditions of the ecological subsystem. Without them, its capacity to provide services cannot be determined. Therefore, we consider ES potentials as an integral part of the state (of the ecosystem) component. Concerning benefits, values, and services, Kandziora et al. (2013, p. 56) proposed to ascribe benefits and values to the impact component (see Fig. 2a), leaving ES as an intermediate step between state and impact. In this context, we argue that ES are a *means* and not *the object of* the impact: A changed state of the ecological conditions we live in impacts on our well-being through a changed provision of ES, and therefore, we allocate ES to DPSIR’s impact component, rather than in between (see Fig. 2b).

Another structural mismatch of the DPSIR and the cascade arises from the strong bipolarity of the cascade, which does not correspond to the standard DPSIRs structure. The logic of the cascade dictates that one end represents the biophysical realm of ecosystems and the other represents societal benefits and values. This double-ended structure is not evident in the DPSIR. The state (of the ecosystem) obviously is part of the natural subsystem, but impacts and responses describe links between nature and society, rather than components of the one or the other. Benefits and values on the other hand clearly are part of societal processes and structures, and we argue they even define the state of the societal subsystem in a SES. Consequently, we propose the introduction of a second state component—the *state of the societal system* (see Fig. 2c). This component represents the societal conditions resulting from an impact that an altered state of the ecosystem has. That impact consists in either a decline or a rise in the delivery of ES, which affects the generation of benefits in the state of the societal system, which in turn triggers responses. Due to the introduction of a second state component, we can now separate drivers that exert pressure on the ecosystem (as in the classic DPSIR) from drivers that exert pressure on the state of the societal system (see Fig. 2d). Analogously, we distinguish societal responses that affect the driver from ecosystem responses, for example, the natural adaptation of ecosystem properties to climatic variability.

To account for the strong human involvement within the ‘cascade process’ as postulated by Spangenberg et al. (2014), we replaced the DPSIR’s response module with a new component called *human involvement* (see Fig. 2d). Human involvement comprises all human activities that influence (i.e., either impair or facilitate) the delivery of an ES: direct alterations to the ecosystem structures and processes (e.g., road construction or chemical pollution), use value and spiritual value attribution, and activities required for the mobilization and appropriation of ES. By introducing this module, we established a second causal chain, connecting the state of the societal system with the state of the ecosystem: Changes in benefits (e.g., a decline in safety from natural hazards), which entail a changed perception of values or preferences, then result in changed human involvement, e.g., an adjustment of forest management. The altered human involvement finally affects and modifies ecosystem properties, their ecosystem functions and/or their ES potentials. This does not have to be a physical process: “With use value attribution, while the biophysical situation is unchanged, its perception is altered” (Spangenberg et al. 2014, p. 25). This means that a changed use value attribution results in altered ES potentials and therefore changed ES delivery, without physically interfering with the ecosystem properties and functions themselves.

**Fig. 3:**
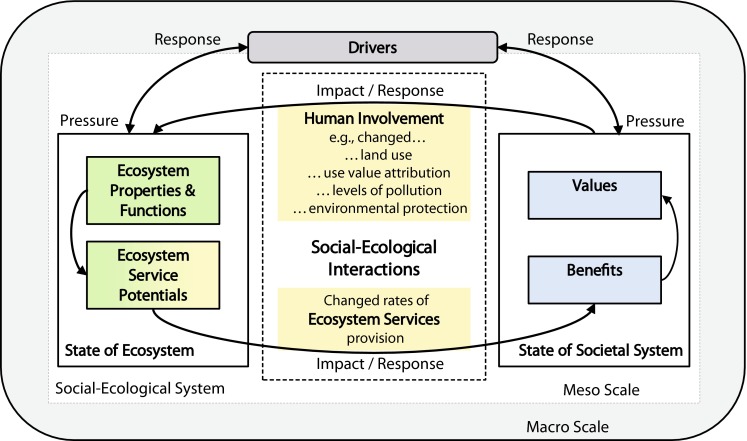
The cycle of ecosystem service provision and societal feedback. Drivers exert pressures both on the state of the ecosystem and/or on the state of societal system, which causes impacts via altered social–ecological interactions (SEIs). Depending on the subsystem exposed to the pressure, the impact is expressed either through altered human involvement or through a changed delivery of ecosystem services. Responses to these alterations either affect the drivers directly, and/or the state of the impacting subsystem via SEIs (e.g., altered use value attribution, land-use changes, or changes in the ES provision)

The separation of the two state modules and the introduction of human involvement entail a new understanding of impacts and responses: Under our new framework, impacts and responses describe the linkages between nature and society in the form of social–ecological interactions (SEIs) (see Fig. 3). These include human involvement and as its ‘counterpart’ the delivery of ES. Thus, many human activities, which the traditional DPSIR attributes to pressures, are now considered impacts. However, our model identifies and quantifies impacts (and responses) without any positive or negative connotation. They are just ‘changes’ in the delivery of ES or ‘changes’ in human involvement. This opens the framework to more differentiated discursive positions.

## Example case: the Río Mecina Valley

We applied our framework for the analysis of the causal relationships within the SES of the *Río Mecina* drainage area (see Electronic Supplementary Material—S1 for a detailed description of the study area and affiliated stakeholders). The objective was to test the frameworks applicability within the boundaries of a well-defined and comparably small-sized geographic area. The problem context consisted of a ‘classic’ dilemma of the Mediterranean mountain SESs: the high proneness to erosion and to forest fires. We centered our ‘test analysis’ on the general focal question: What characterizes the past and current SEIs (i.e., human involvement and provision of ES) and how do they shape the ecosystem and the social system in the *Río Mecina* drainage area? What are the natural and human-induced factors that initiate changes and lead the behavior of the SES in the valley? What are possible future pathways for that system?

**Fig. 4:**
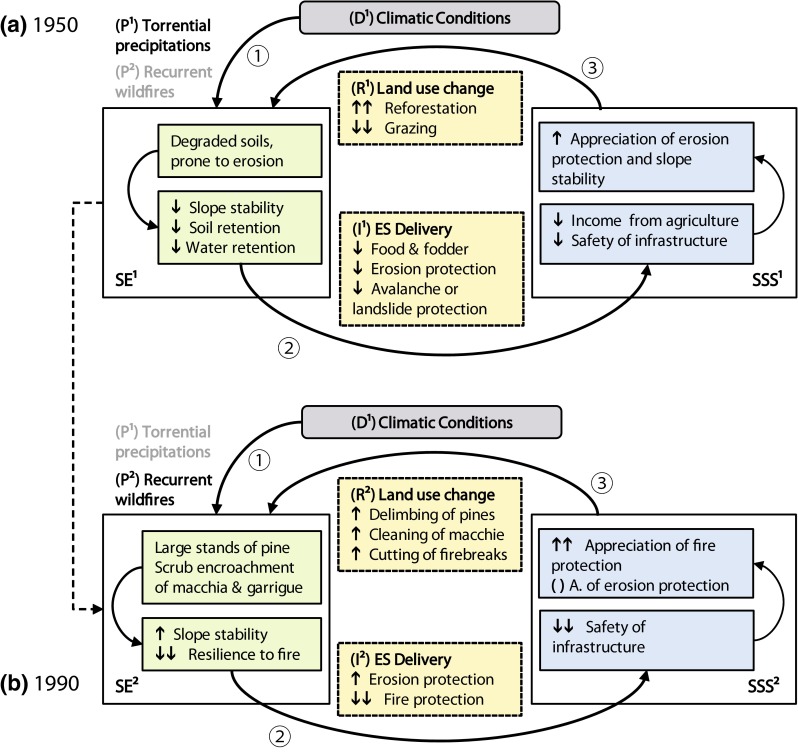
Graphic representation of the past development of the study area, related to the influence of the driver ‘climatic conditions’ (*gray boxes*). The *numbers (1)*–*(3)* indicate the causal sequences Pressure (1) ➔ State ➔ Impact (2) ➔ State ➔ Responses (3) at two different points in time (i.e., shortly before and well after the reforestation measures in the 1960s and 1970s). Pressures, impacts, and responses are affecting the system simultaneously and constantly, yet their intensity gradually changes over time. The *dashed arrow* indicates this gradual change over time of both, the state of the ecosystem and of the social system. *Green*-*colored boxes* indicate components of the state of the ecosystem (SE): ecosystem properties and functions (*upper box*) and ES potentials (*lower box*). *Blue*-*colored boxes* indicate components of the state of the societal system (SSS): benefits (*lower box*) and values (*upper box*). *Yellow colors* indicate social–ecological interactions: changed ES delivery (*lower box*) and changed human involvement as a response to it (*upper box*). The *arrows* ↑/↓ indicate an increase/decrease in intensity. ( ) indicates an unchanged intensity

**Fig. 5:**
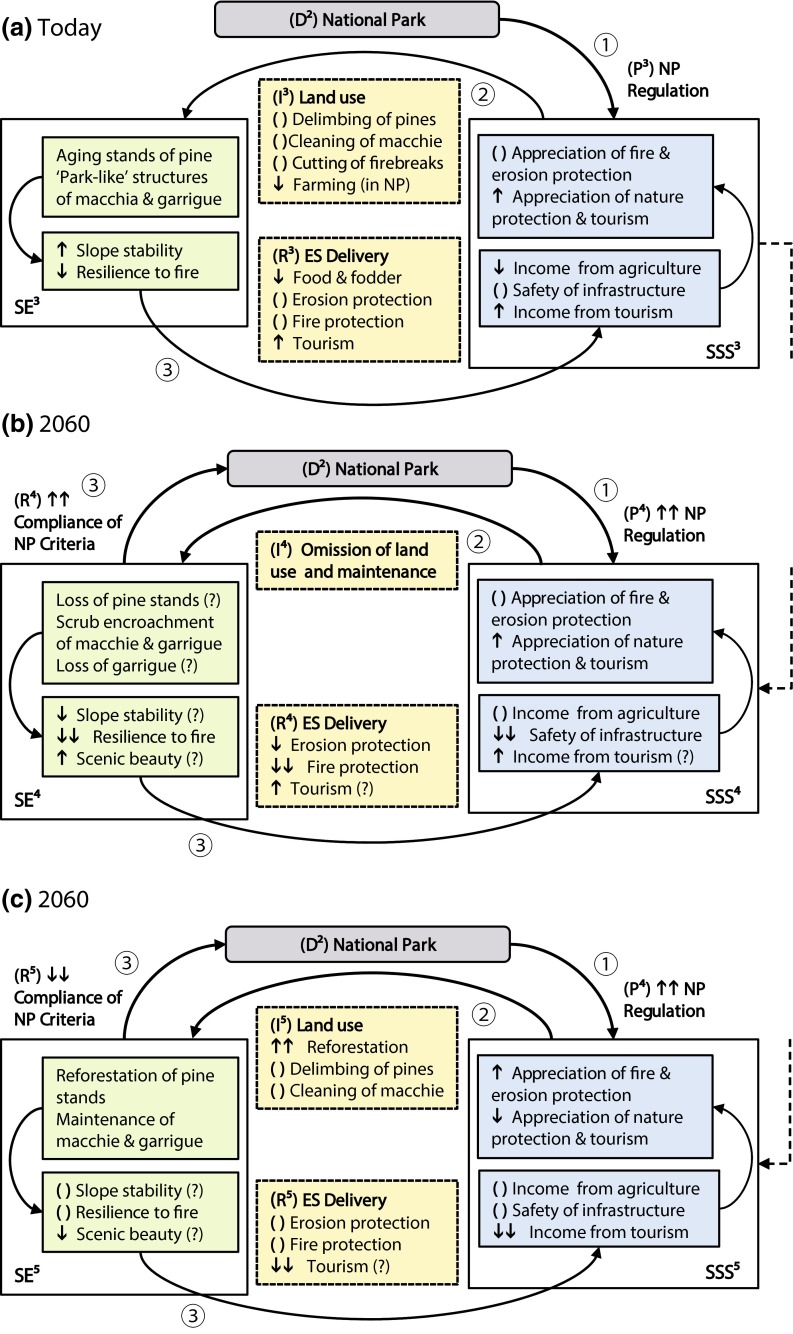
Two possible pathways for the future development of the study area under the increasing influence of the driver ‘National Park’ (*gray boxes*) and its regulations (P). The *numbers (1)*–*(3)* indicate the causal sequences Pressure (1) ➔ State ➔ Impact (2) ➔ State ➔ Responses (3) today (**a**) and two possible future situations in 2060 (**b**, **c**). These two scenarios assume either a rigorous enforcement of the NP regulations (**b**) or “business-as-usual” with continued strong human intervention in the area of the NP. *Green*-*colored boxes* indicate components of the state of the ecosystem (SE): Ecosystem properties and functions (*upper box*) and ES potentials (*lower box*). *Blue*-*colored boxes* indicate components of the state of the societal system (SSS): benefits (*lower box*) and values (*upper box*). *Yellow colors* indicate social–ecological interactions: Changed human involvement (especially land-use) (*upper box*) and a changed ES delivery as a response to it (*lower box*). The *arrows* ↑/↓ indicate an increase/decrease in intensity. ( ) indicates an unchanged intensity, and *question marks* indicate a questionable prediction

In the course of preparation, we consulted scientific literature, publicly accessible policy and statistical documents, reports from local organizations, touristic brochures, and websites. In the following, we used questionnaires and conducted semi-structured interviews with the local population and tourists to evaluate their perceptions, opinions, and concerns. We conducted expert interviews with the administration staff of the Sierra Nevada National Park as well as staff of the local and regional administration, to gain insights into, e.g., administrative objectives, legal structures, or interest conflicts. Our last target group was (environmental) scientists researching in the study area. Furthermore, we complemented these social-scientific approaches, with compiled results of own preceding and ongoing ecological research (e.g., climate data, soil sampling, dendrology). Based on this preparatory work, we chose two drivers of change to serve as the starting points for our exemplary analysis: the prevalent climatic conditions and the establishment of the Sierra Nevada National Park (IUCN category II) and its consequences for the valley. From these starting points, we followed the causal sequences established by our framework, to acquire a preliminary, qualitative, and multi-temporal causal scheme. To visualize the manner of functioning of our framework, we graphically represent the results of our analysis, in a simplified and generalized form, in Figs. 4 and 5. While Fig. 4 illustrates the past development of the study area, Fig. 5 shows two possible pathways for future development.

### Driver 1: Climatic conditions

The first driver (D^1^) we chose for analysis, the prevalent ‘climatic conditions,’ exerts two main pressures defined by its major characteristics (see Fig. 4a, b): year-round aridity, facilitating recurrent wildfires (P^2^), and torrential precipitations in winter (P^1^), which are causing high levels of pluvial erosion. Especially, since the departure of the Moors, these pressures have been increasingly altering the ecosystem properties, which underlie the state of the ecosystem (SE^1^). Decreasing slope stability, increasing formation of gullies and soil loss, and accordingly a reduced water retention capacity are the consequence—a development, well known in Mediterranean mountain systems. The impact (I^1^) of the degraded ecosystem expressed in the decline of a number of ES, among which our interviewed experts and locals considered reduced erosion control, reduced provision of food and fodder, reduced water quality regulation, and the reduced water provision the most important ones. The consequent regression in ecosystem benefits led to a changed perception of values: Reduced income from agriculture and a decline in the safety of infrastructure and property were the two major reasons for a ‘rethinking’ of the societal value of ES potentials such as slope stability, soil retention capacity, and water retention capacity. To local farmers, these potentials were valuable all along, yet the severity of the occurring erosion and degradation and their economic consequences induced the local and regional governments to take action as well. Ultimately, this caused two sets of responses (R^1^): It triggered the reforestation measures undertaken in the 1960s and 1970s, but it also contributed to encourage the migration from the area, leading to less sheep and goat grazing.

Since the 1970 s, the responsive measures (R^1^) gradually altered the prevalent ecosystem properties (SE^2^). Migration from the area helped to decrease grazing, initiating the formation of garrigue and advancing the succession of former garrigue areas to macchia. In addition to that, wherever reforestation was successful, closed stands of pine forest exist today. The altered ecosystem properties and functions enhanced ES potentials such as slope stability and soil quality and—together with the increased acknowledgement of their importance by local and regional stakeholders—intensified the provision of the ES erosion control, provision of food and fodder, and others (I^2^). Yet scrub encroachment and reforestation with pines also substantially increase the area’s vulnerability to fires (Trabaud 1976). The presence of the pine forest stands in the valley further exacerbates this ‘natural’ proneness to fire (Corona et al. 2014). The modified state of ecosystem (SE^2^) today delivers a differentiated set of ES: On the one hand, erosion control and other services affiliated with soil stability increased, yet fire protection substantially decreased (I^2^). Responses (R^2^) to the decreased delivery of fire protection are in evidence in the field. These include the debranching of pine trees up to a height of 2 m, clearing out dead wood, cutting vast firebreaks into the stands, and the extraction of all shrubs except certain endemic species in the macchia formations, creating almost park-like structures.

### Driver 2: Establishment of the National Park

With the declaration of the Sierra Nevada National Park in 1999 a new driver came into operation: the National Park (D^2^). This driver does not exert any direct pressures on the state of the ecosystem. Instead, it affects the state of the societal system (SSS^3^) through its rules and regulations (P^3^), which then result in changed land use and management (see Fig. 5). It changes access rights and renders new sources of income accessible (e.g., eco-tourism), while obstructing others (e.g., hunting tourism). The perception of these changes by the local population is ambivalent. While criticizing specific rules and regulations, most of the interviewed locals spoke out in favor of the NP in general. They appreciate species and biodiversity protection, yet their main concern is the conservation of the cultural landscape as the basis of their income from agriculture and tourism. Thus, local stakeholders valued fire and erosion protection as well as water provision the highest.

The regulations of the NP ban any exploitation or management practices that interfere with its protection objectives, as category II protected areas are defined to be natural systems or at least in the process of being restored to natural systems (Dudley 2008). This means that, in theory, the category II status does not provide for extensive interventions (I^3^) such as the large-area extraction of ‘unwanted’ macchia species or the debranching and cleaning from deadwood of whole pine stands. Yet without these measures, the hazard to the ecosystem and to the safety of the local population by wildfires is not tenable.

Another major conflict will arise when the pine plantations reach their maximum stand ages: Regeneration is almost null in plantation with densities over 1500 pines/ha and still very low in stands with moderate densities (Gómez-Aparicio et al. 2009). Consequently, the very even-aged plantations in the research area are likely to reach maximum ages in the same, rather small, time window. To maintain these stands and their important ecosystem functions, great efforts will become inevitable. In summary, this means that on the one hand without extensive human intervention, these areas will increasingly be exposed to high risk, yet on the other hand, these interventions compromise the main objective of the NP, namely the transition to an undisturbed natural system. Figure 5 visualizes this conflict: The first scenario (Fig. 5b) assumes, that according to a strict interpretation of NP regimentations (P^4^), all major human intervention in the NP is omitted (I^4^). According responses (R^4^) include on the one hand the compliance of the state of the ecosystem with the idea of a NP as an area in the transition to ‘untouched wilderness,’ yet on the other hand, the delivery of ES such as ‘fire protection’ will probably be severely constrained (with all consequences for the state of the societal system). Under scenario 2 (Fig. 5c) the maintenance of macchia, garrigue, and the pine forests is continued and reforestation measures are initiated to ensure slope stability (I^5^). Responses (R^5^) to this impact are equally ‘double-edged’ as in the first scenario: On the one hand, erosion protection and fire protection continue at the same level, yet on the other hand, the areas compliance with NP requirements is at least questionable, which might jeopardize its NP status.

## Discussion

In the introduction to this paper, we summarized some major critiques of the DPSIR, the ES concept in general and the ES cascade in particular. In order to address these points of criticism, we developed our framework by merging and modifying the DPSIR and the ES cascade. In the following, we wish to first discuss the need for and the added value of the framework derived from this merging and then compare it to several frameworks with a similar scope.

### Why merge the DPSIR and the cascade?

1. Using merely one of the two frameworks does not capture the whole problem complex and does not satisfyingly answer our focal questions.

A merely ES-based analysis of the research area would not have captured the whole problem complex. The ES cascade describes important factors such as causes of land-use change rooting in the societal system (e.g., migration from the valley) or societal feedback mechanisms like the adaptation of land-use (e.g., afforestation with pines) are not sufficiently conceptualized under the ES cascade. An analysis of our example case based solely on the ES cascade would therefore have meant to view, SEIs such as land-use merely through the lenses of its (direct) influence on ecosystem service delivery—leaving its causes unexplained. Thus, our initial objective for the fusion of the DPSIR and the ES cascade was to embed the cascade into a broader SES framework and thereby enhance its applicability and scope. We chose the DPSIR as ‘counterpart’ for the ES cascade for two main reasons: Firstly, the DPSIR identifies and describes the causes and effects of human-induced changes to the environment, whereas the ES cascades causally link the environment and its structures to the fundaments of society. Thus, they each conceptualize one of the two reciprocal linkages between the essential subsystems of a SES. Secondly, the ‘causal cycle logic’ of the DPSIR allows expanding the unidirectional causal chain of the cascade to include causes of change to the SES, their consequences, and feedbacks.

Just as the ES cascade is an unsatisfying tool to capture the problem complex of our research area, the DPSIR alone would not have sufficiently comprised all necessary aspects. Of course, when using the DPSIR, nothing limits one from complexifying particular problem areas or including both social and natural drivers and complex environmental state changes that happen in multiple phases. In its standard form, however, it is too much geared to human-induced problems and societal responses (Svarstad et al. 2008; Potschin 2009; Kelble et al. 2013). Under a classic interpretation of the DPSIR and the common definitions of its components, the establishment of the NP would not be considered a driver of change to the system rather than a response to environmental problems. Yet, regarding a NP merely as a responsive measure risks underrepresenting possible ‘negative’ impacts of a rigorous enforcement of its regulations. In our case, the omission of land-use in the NP helps species and biodiversity protection, yet it also increases the risk of forest fires. Moreover, even if the establishment of the NP is considered a driver, the standard DPSIR only allows for direct (negative) effects on the environment to ‘count’ as pressures, which means to blind out any pressure the NP exerts on the societal subsystem. In our view, it is essential to include anthropogenic and ecological contributions to an environmental problem’s generation or aggravation equally. Spangenberg et al. (2015) emphasize this shortcoming of the standard DPSIR and propose to remedy it by combining two DPSIR cycles—a social and an ecological cycle. The same principle is implemented in our framework: We include one possible causal cycle originating in the biosphere (see Fig. 4), as well as a possible cycle initiated by pressures within the societal realm (see Fig. 5).

2. Combining the two frameworks can eliminate several of their individual conceptual flaws.

The ES concept is often criticized for its anthropocentric focus (McCauley 2006; Sagoff 2008). Several scholars warn that regarding nature merely as a provider of societal benefits promotes an exploitative human–nature relationship and encourages the commodification of nature (McCauley 2006; Fairhead et al. 2012; Turnhout et al. 2013). However, we argue, by including reciprocal feedbacks between nature and society, the combination of the DPSIR and the cascade alleviates this bias. The cycle of ecosystem service provision and societal feedback interprets ES as the outcome of protective as well as exploitative human involvement. Human involvement is the result of manifold societal decision and negotiation processes. In the consequence, the delivery of one ES is often a trade-off to the detriment of another. Therefore, one stakeholder may consider the provision of a service positive, while a different stakeholder considers it negative as it supersedes other services. This also directs at the criticism against the normative nature of the ES concept, allegedly considering all outcomes of ecosystem processes desirable (see e.g., McCauley 2006). ES underpin human well-being and are therefore in principal desirable, yet this is strongly dependent on a stakeholder’s aims and needs. However, the concept of ES helps identify, compare, and evaluate these differing societal aims and needs in SES, by comparing and valuing different ES. Thus, by including ES, our approach enables the analysis of societal trade-offs and the formulation of management goals. Thereby, it addresses another point of criticism mentioned above: The inability of the DPSIR, to encourage proactive management, due to its focus on environmental problems and consequential responses as pointed out by Kelble et al. (2013).

While ES are often criticized for their anthropocentric orientation, the DPSIR is contested for its biocentric focus. Svarstad et al. (2008) criticize traditional applications of the DPSIR for representing primarily the ‘Preservationist’ discourse without capturing the necessary information for differing discourse types, such as the ‘Traditionalist,’ ‘Win–Win,’ or ‘Promethean.’ For example, they point out that the Traditionalist discourse type’s focus lies not on the state of the ecosystem, “but instead on the state of social matters” and its perspective is therefore not accounted for in the standard DPSIR. Furthermore, the ‘Traditionalist’ is most concerned with impacts on (local) people, rather than impacts on the ecosystem—another discursive approach that the classic interpretation of the DPSIR disregards. We argue that by introducing the second state component and by including the ES cascade, we open our framework to a variety of stakeholder perspectives that the original DPSIR does not account for.

### Comparison with other frameworks

The framework presented here is centered on the question, how we can analytically describe the interactions between nature and society that shape these systems. Binder et al. (2013) reviewed and compared ten frameworks for analyzing SES—including the DPSIR and ES. Only three out of ten frameworks “address the reciprocity between the social and the ecological systems” (Binder et al. 2013, p. 35)—a criterion we regard as essential for the analysis of SES and that was therefore emphasized in our framework. This also holds true for the “option to treat the social and ecological systems in almost equal depth” (Binder et al. 2013, p. 37), as our framework represents both subsystems equally well and in equal depth. Despite the importance of this prerequisite, Binder et al. (2013) found that only one framework provides this option: Elinor Ostrom’s SES framework (SESF) (Ostrom 2009; McGinnis and Ostrom 2014). Our framework corresponds with several other characteristics of Ostrom’s SESF. However, there are also distinct differences. The SESF separates the subsystems ‘resource system’ from ‘resource units’ and simply describes the latter to be “be part of” the former (Ostrom 2009; McGinnis and Ostrom 2014), while our framework uses the causal logic of the ES cascade to explain how ES (‘resource units’) emerge from ecosystem structures and processes (‘resource system’). This exemplifies a different focus: Our framework focuses more explicitly on analyzing the *causal linkages* between the system variables. The SESFs central interface, called *Focal Action Situations*, describes how “inputs are transformed by the actions of multiple actors into outcomes” (McGinnis and Ostrom 2014, p. 34); thus, the SESF follows a kind of ‘input–output logic’: All subsystems contribute a certain input that is than transformed into outcomes and feeds back on four different subsystems. Our framework, on the contrary, follows the logic of a causal cycle consisting of reciprocal impacts between two subsystems. All in all, the SESF has a broader scope and a more open structure that allows for other frameworks and models—including the one presented here—to be integrated (Binder et al. 2013; McGinnis and Ostrom 2014).

## Conclusion

This paper shows that the DPSIR as well as the ecosystem service cascade, if used separately, can only capture parts of the complex interactions in real-life SES. Even if applied consecutively, such analyses cannot generate the causal sequences required for the understanding of SEIs in a SES. Our approach, to close the cycle of ecosystem service provision and societal feedback, took this task one step further. Here, we include the causes of change to SES, reproduce their effects and their consequences, and express these cause–effect relationships. In doing so, our approach facilitates an evaluation with the methodologies of ES analyses. Thus, it connects to the growing body of work on ecosystem service measurement and valuation, which makes it accessible to a broad spectrum of scientists, environmental planners, and policy makers. Heretofore, we tested the framework for the first time. Further application will prove its validity and usefulness regarding various research questions, different spatial and temporal scales, and diverse regional contexts. As such, the new approach will stimulate the continuous debate over and search for applicable frameworks for the analysis of SES.

## Electronic supplementary material

Supplementary material 1 (PDF 478 kb)
