# Supplementary material for: Ecosystem services in coupled social–ecological systems: Closing the cycle of service provision and societal feedback
Source: Ambio. 2015 May 12;44(8):737–49. doi: 10.1007/s13280-015-0651-y (PMC4646852; doi:10.1007/s13280-015-0651-y)
Supplement: Supplementary file 1 — Supplementary material 1 (PDF 478 kb) [file 13280_2015_651_MOESM1_ESM.pdf]

AMBIO

Electronic Supplementary Material
This supplementary material has not been peer reviewed.

Title: **Ecosystem services in coupled social–ecological systems—closing the cycle of service provision and societal feedback**

Authors: Michael Nassl, Jörg Löffler

Study area

The Río Mecina drainage area (~46 km^2), which largely coincides with the borders of the municipality Alpujarra de la Sierra, is located north of the village Mecina Bombarón (36°59'N, 3°09'W), within the southern declivity of the Spanish Sierra Nevada mountain range. In 1986, UNESCO declared the Sierra Nevada a Biosphere Reserve. In 1999, the center became a National Park (IUCN category II), with a surrounding Natural Park, which already exists since 1989 (Junta de Andalucia 2014). The southernmost geological boundary of the Río Mecina valley coincides with the border of the Natural Park, whereas its northern parts are located in the National Park (NP). Typical for the Mediterranean climate, aridity characterizes the prevalent climatic conditions: Hot and very dry summers alternate with winters bringing seldom, yet mostly torrential precipitation (Carrion et al. 2009). Following widespread deforestation of the region during the Roman Age, the Moors installed extensive irrigation agriculture, consisting of terraced fields and a ramified system of irrigation ditches (acequias) (Seuffert 2000). With the departure of the Moors, their terrace system gradually decayed and, in combination with extensive goat and sheep grazing, erosion became one of the major problems in the valley. To face the severe soil erosion and damage to infrastructure and property, large-scale reforestation measures were taken mainly from 1960 to 1980 (Gómez-Aparicio et al. 2009). The historic land-use left a patchwork of remaining, potentially natural stands of holm oaks (Quercus ilex), large reforested stands of pines (Pinus halepensis, Pinus pinaster, Pinus nigra and Pinus sylvestris), as well as patches of macchia and garrigue scrubland, interrupted by degraded, bare soil grounds and agricultural lands.

Stakeholders

In our example case, we focused on three target stakeholder groups: The local population (i.e., farmers, shepherds and villagers), international and national tourists and environmental authorities (i.e., NP rangers and administration, forestry commission, regional authorities).

Local population

The population development in the study area reflects the general development of rural areas in the Sierra Nevada. Beginning in the 1950s, structural transformation and the associated loss of importance of the primary sector, facilitated by European emigration conditions and the increasing degradation of soils in the Sierra Nevada, lead to a strong emigration to economically better-off regions in Spain and Central European countries (Cózar Valero 2000). The population in the municipality Alpujarra de la Sierra decreased by almost two thirds between 1950 and 2011. Today it comprises a population of 1140 with a population density of 16.5 habitants per km^2 (Instituto de Estadística de Andalucía 2011). Due to this migration and a decline in birth

rates, the population structure in the research area is characterized by overageing: While only about 10% are under 15, almost one third (27,55%) of the population is more than 64 years old (Instituto de Estadística de Andalucía 2011). Unemployment rates over 30% further exacerbate this pressure on the income generating parts of society. The two main sources of income in the Alpujarra de la Sierra are agriculture and tourism.

Agriculture in the area mainly comprises smallholdings (minifundios). The Censo Agrario 2009 counts 94 total agricultural holdings, of which 75 own less than 10 ha (Instituto de Estadística de Andalucía 2009). The majority of these smallholdings raise crops, especially beans and peas, or raspberries. About one third of the agricultural holdings (additionally) practices pasture farming (Instituto de Estadística de Andalucía 2009). Pastoral farming consists mainly in transhumance (i.e., the seasonal movement of people with their livestock between higher summer and lower winter pastures), yet traditional animal husbandry is increasingly in decline in the region. Results from our interviews showed strong concerns regarding the degradation of the cultural landscape amongst farmers and shepherds in the study area. In their view, the abandonment of traditional terrace agriculture and transhumance and the loss of traditional knowledge pose the greatest problem. The main reason for this can be found, according to them, in the low profitability and the consequential low attractiveness for the younger generation. Accordingly, many interviewees mentioned overageing and migration as major problems. Tourism has become the main source of income in the Río Mecina valley. It profits from the proximity to the highly touristic coast as well as from the recreational potential of the Sierra Nevada. Fifteen casas rurales (guesthouses or small hotels) provide around 230 sleeping accommodations in the municipality (Ayuntamiento de Alpujarra de la Sierra 2014). Apart from the accommodation business itself, the sale of local products (e.g., honey, Serrano ham or pottery) create additional revenue. The municipality has three traditional butcheries and four small shops selling, amongst other things, local souvenirs (Ayuntamiento de Alpujarra de la Sierra 2014). In many cases, a secondary occupation in the primary sector or in the building sector, either in or outside of the valley complements the family income from tourism. Several of the interviewed villagers expressed concerns at the increasing dependence on tourism and possible environmental consequences, especially from housing sprawl and road construction. Nonetheless, promoting and expanding tourism in the valley is regarded as the most promising future development.

Tourists

Tourists in the area include international tourists on the one hand and Spanish tourists on the other. As our interviews have shown, regional guests mainly visit the Alpujarra for one night or for the weekend. Their focus lies on enjoying local products (i.e., wine and ham), socializing and experiencing traditional ‘village life’, whereas outdoor activities like hiking only slowly become a

priority for Andalucian tourists. International tourists usually stay longer and mainly pursue nature activities like hiking, mountain biking or trail riding - they come to 'experience nature'. However, nature tourism in the area is still of minor importance, but it shows an upward trend.

Environmental Authorities

The upper parts of the study area fall within the jurisdiction of the National park, while the lower valley lies in the Natural Park. Thus, both areas fall under different protection regimes. In accordance with the Andalucian environmental protection laws, the Natural Park is geared towards environmentally sustainable rural development (ecodesarollo) and focuses on the improvement of the living conditions for the local population and nature protection alike. The National Park, by contrast, is legally bound to the regimentations of an IUCN category II protected area and therefore its predominant goal is the re-establishment of ecological integrity and biodiversity protection. While the objectives of the Natural Park largely coincide with the values and aims of the local population, the relationship between the National Park and other stakeholders is often problematic. Locals acknowledge species and biodiversity protection and welcome the positive effect on tourism that the NP has in their eyes, however they criticize the NP's assumed 'ecology-first dogma'.

References

Ayuntamiento de Alpujarra de la Sierra. 2014a. Casas rurales en Mecina Bombarón, Yegen y El Golco. Retrieved 1 October, 2014, from http://www.alpujarradelasierra.es/casas-rurales (in Spanish).

Ayuntamiento de Alpujarra de la Sierra. 2014b. Comercios. Retrieved 1 October, 2014, from http://www.alpujarradelasierra.es/comercios (in Spanish).

Junta de Andalucia. 2014. Nature Area Sierra Nevada. Retrieved 22 May, 2014, from http://www.juntadeandalucia.es/medioambiente/servtc5/ventana/mostrarFicha.do?idEspacio=7418&lg=EN

Seuffert, O. 2000. Von der Kultivierung zur Degradierung der Landschaft im Mittelmeerraum. *Petermanns Geographische Mitteilungen* 144/6: 36–51 (in German).

Carrion, J.S., S. Fernández, G. Jiménez-Moreno, S. Fauquette, G. Gil-Romera, P. González-Sampériz and C. Finlayson. 2009. The historical origins of aridity and vegetation degradation in south eastern Spain. *Journal of arid environments* 74: 731–736.

Gómez-Aparicio, L., M.A. Zavala, F.J. Bonet and R. Zamora. 2009. Are pine plantations valid tools for restoring Mediterranean forests? An assessment along gradients of climatic conditions, stand density and distance to seed sources. *Ecological Applications* 19: 2124–2141.

Cózar Valero, M.E. 2000. Los recursos humanos en un espacio natural protegido: Sierra Nevada. *Cuadernos Geográficos* 30: 325–346 (in Spanish).

Instituto de Estadística de Andalucía. 2009. Censo Agrario 2009. Resultados de Andalucía. Retrieved 1 October, 2014, from http://www.juntadeandalucia.es/institutodeestadisticaycartografia/iea/consultasActividad.jsp?CodOper=703&sub=45376 (in Spanish).

Instituto de Estadística de Andalucía. 2011. Censos de Población y Viviendas 2011. Retrieved 1 October, 2014, from

http://www.juntadeandalucia.es/institutodeestadisticaycartografia/institutodeestadisticaycartografia/censo2011/index.htm (in Spanish).
